# Laparoscopic Treatment of Type III Mirizzi Syndrome by T-Tube Drainage

**DOI:** 10.1155/2016/1030358

**Published:** 2016-05-15

**Authors:** Fahri Yetışır, Akgün Ebru Şarer, H. Zafer Acar, Yılmaz Polat, Gokhan Osmanoglu, Muhittin Aygar, A. Erdinc Ciftciler, Omer Parlak

**Affiliations:** ^1^General Surgery Department, Private Minasera Aldan Hospital, 06810 Ankara, Turkey; ^2^Anesthesiology and Reanimation Department, Atatürk Research and Training Hospital, Ankara, Turkey; ^3^General Surgery Department, Natomed Private Hospital, Ankara, Turkey; ^4^General Surgery Department, Fırat University, Elazığ, Turkey; ^5^General Surgery Department, Medical Park Private Hospital, Ankara, Turkey

## Abstract

Mirizzi syndrome (MS) is an impacted stone in the cystic duct or Hartmann's pouch that mechanically obstructs the common bile duct. We would like to report laparoscopic treatment of type III MS. A 75-year-old man was admitted with the complaint of abdominal pain and jaundice. The patient was accepted as MS type III according to radiological imaging and intraoperative view. Laparoscopic subtotal cholecystectomy, extraction of impacted stone by opening anterior surface of dilated cystic duct and choledochus, and repair of this opening by using the remaining part of gallbladder over the T-tube drainage were performed in a patient with type III MS. Application of reinforcement suture over stump was done in light of the checking with oliclinomel N4 injection trough the T-tube. At the 18-month follow-up, he was symptom-free with normal liver function tests.

## 1. Introduction

Mirizzi syndrome (MS) was first described in 1948, as a repeated inflammation of the gallbladder due to an impacted gallstone in the cystic duct or Hartmann's pouch which may cause intermittent or constant mechanical obstruction in the common bile duct (CBD) [1]. The pathophysiological process leading to the subtypes of MS has been explained as an inflammatory phenomenon secondary to a pressure ulcer caused by an impacted gallstone which can cause first external obstruction of the bile duct and eventually erodes into the bile duct evolving into a cholecystocholedochal fistula with different degrees of communication between the gallbladder and bile duct [2, 3]. Csendes MS classification system has been renewed by Beltran in 2012; depending on the degree of involvement of the biliary tract, the patients may be grouped into five distinct groups; MS with coexistence of cholecystoenteric and cholecystocholedochal fistula was accepted as type V [2, 4, 5].

Symptoms are similar to those of acute and chronic cholecystitis, with or without jaundice [1]. In spite of intermittent symptoms, clinical presentation of MS can be outlined in 4 possibilities: obstructive jaundice, history of acute cholecystitis and/or cholangitis, and acute abdomen due to biliary or asymptomatic peritonitis. Diagnosis of MS is not so easy and in some cases diagnosis can be only done during operation. Although there is no consensus about diagnostic choice of MS, all noninvasive methods (Abdominal Ultrasonography (US), computed tomography (CT), and magnetic resonance cholangiopancreatography (MRCP)) have to be used firstly and then if required invasive intervention endoscopic retrograde cholangiopancreatography (ERC), percutaneous transhepatic cholangiography (PTC), and even laparoscopy can be used [6]. Identification of MS before surgery or at least during surgery is important because of the serious morbidity and mortality related to the chronic biliary tree inflammation and anatomic alteration of the bile duct which necessitate a rigorous surgical technique [7]. Correct surgical approach and management are very important for MS in order to decrease the morbidity and mortality [8].

Prevalence of MS among all cholecystectomies is 0.3% to 5.7%. MS is seen especially in female patients with advanced age [2]. MS type I is fairly common (10.5% to 51%) and types II and IV are rather uncommon. The treatment of MS > type II with extensive destruction of the bile duct wall consists of bilioenteric anastomosis. Roux-en-Y hepaticojejunostomy is preferred [4]. Despite the high progress in technology and laparoscopic surgery, open surgery is usually preferred instead of laparoscopic surgery to treat MS with > type II.

We would like to report laparoscopic subtotal cholecystectomy (SC), extraction of impacted stone by opening anterior surface of dilated cystic duct and choledochus, and repair of this opening by using the remaining part of gallbladder over the T-tube drainage in a patient with type III MS.

## 2. Presentation of Case

A 75-year-old man was admitted to the emergency department with the complaint of abdominal pain, intermittent fever, and jaundice for the last six days. In particular, pain was present at the right upper quadrant. In his past history, there was not any operation or chronic disease. Abdominal US and CT showed that so many calculi were present in gallbladder and a large calculus of approximately 2 cm located between CBD and cystic duct and proximal extrahepatic and intrahepatic biliary dilation was seen. He was hospitalized for cholelithiasis and choledocolithiasis in the gastroenterology department. ERC was performed and endoscopic papillotomy was applied and impacted calculus was seen. ERC has been applied twice but this impacted calculus could not be extracted and a plastic stent was placed. The patient was assessed as MS and was transferred to the general surgery department for surgical treatment.

His vital parameters were as follows: blood pressure (BP) 140/90 mmHg, heart rate (HR) 84, and fever 38.2°C. On his abdominal examination, there was evidence of icterus, and sensitiveness was positive at right hypochondrium. In biochemical analysis, total bilirubin was 4.8 mg/dL (normal < 1.0) conjugated bilirubin was 2.9 (normal < 0.2 mg/dL), serum glutamic oxaloacetic transaminase (SGOT) was 66 U/L (5–40 U/L), serum glutamic-pyruvic transaminase (SGPT) was 68 U/L (normal 5–40 U/L), alkaline phosphatase was 247 U/L (<106 U/L), glutamyl transferase was 330 U/L (<45 U/L), LDH was 267 U/L, and total blood count WBC was 11.000 K/UL. He underwent laparoscopic operation under general anesthesia.

## 3. Surgical Technique

**Figure 1:**
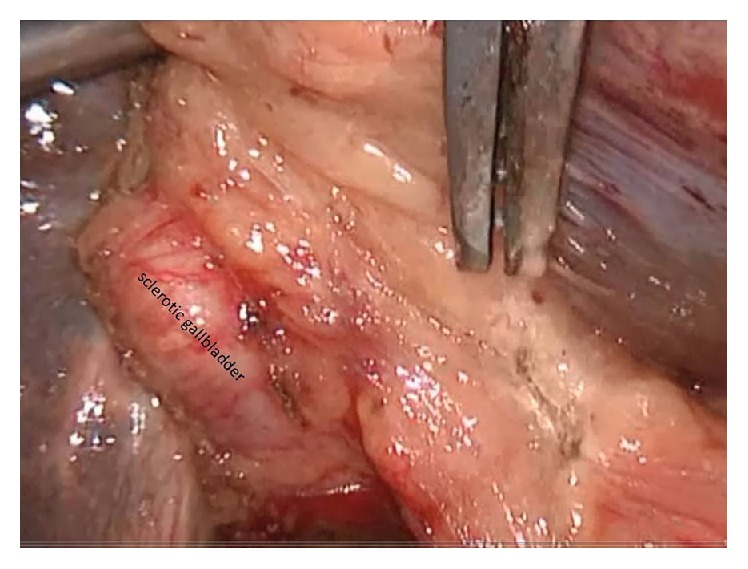
Sclerotic gallbladder is seen.

**Figure 2:**
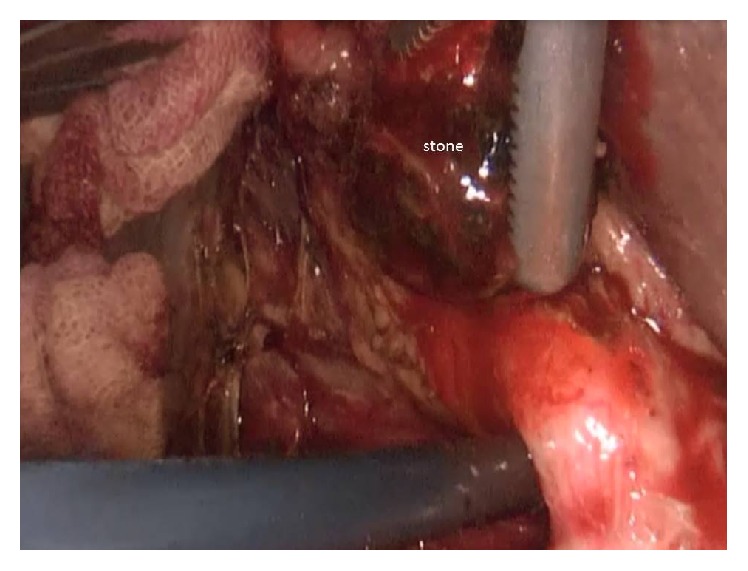
Extracted stone is seen.

**Figure 3:**
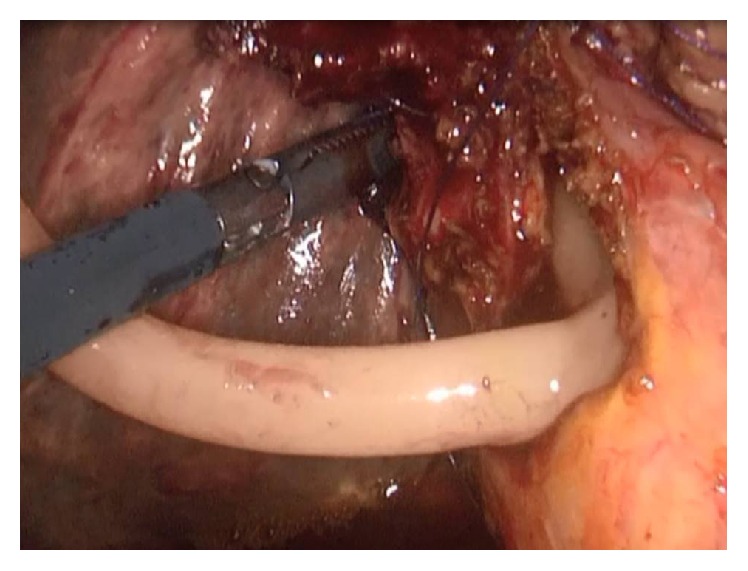
Placed T-tube drainage into the choledochus through the opening after stone extraction and irrigation of choledochus.

**Figure 4:**
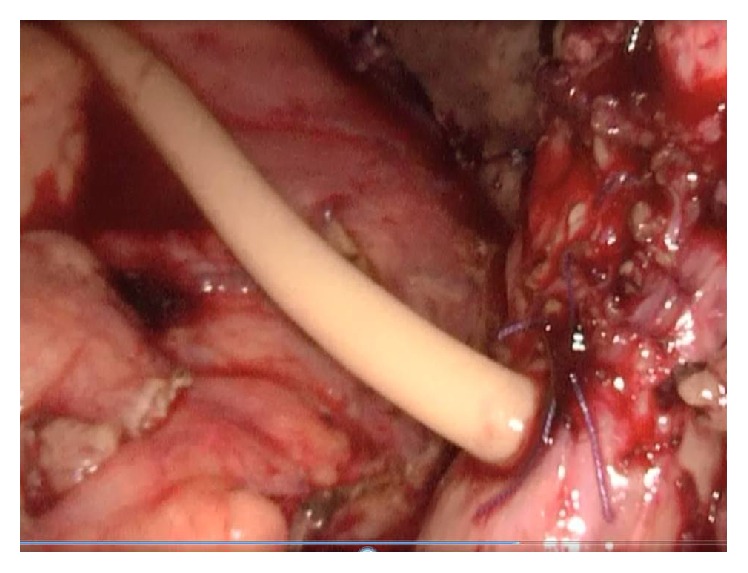
Gallbladder stump and choledochus were repaired over T-tube drainage with interrupted suture.

**Figure 5:**
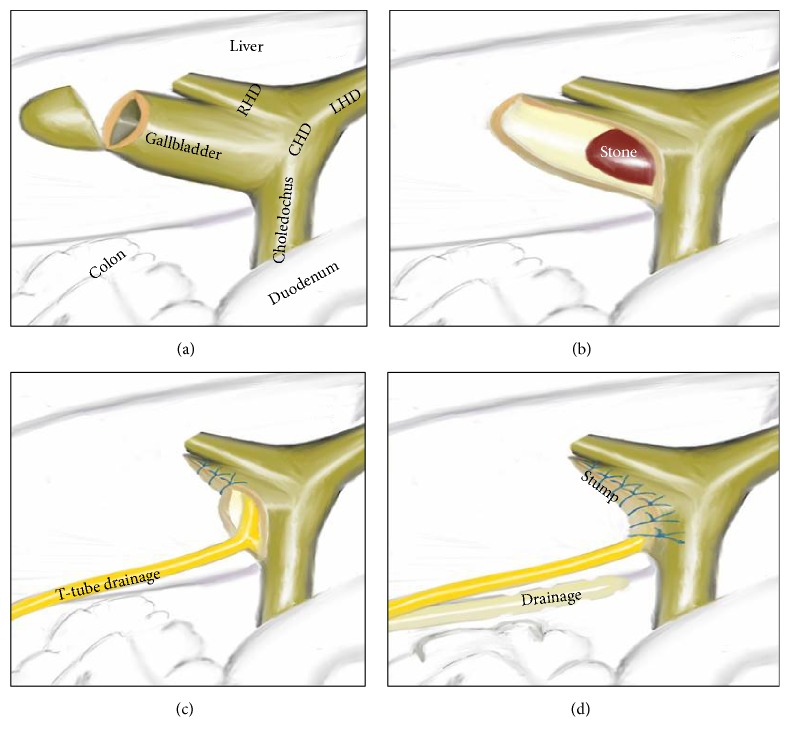
Schematic illustration of this operation. (a) Fundus resection of sclerotic gallbladder. (b) Impacted stone is seen after resection of anterior part of gallbladder and cystic duct. (c) T-tube drainage was placed in choledochus through the anterior opening. (d) Gallbladder stump and choledochus have been repaired over T-tube drainage.

Antibiotic prophylaxis was given during anesthesia induction. Classical 4 trocars entries were used. There were dense adhesions between colon, duodenum, gallbladder, and omentum in the right subhepatic space. After gentle dissection, all the anatomical structures were separated from each other. Sclerotic shrunken gallbladder with distorted anatomy with frozen Calot's triangle was observed (Figure 1). Later, there were significant difficulties in dissecting the gallbladder neck and Calot's triangle and further dissection would expose the patient to a higher risk of common bile duct injury or hemorrhage. Fundus (fist) dissection had to be applied instead of Calot's triangle. After dissection, the fundus was opened and inside approach technique of Hubert et al. was used [9]. All gallstones were extracted one by one through this incision in the fundus. Impacted stone could not be extracted from this opening. Incision on the anterior surface of gallbladder was extended all through the cystic duct and an anterior part of choledochus near the cystic duct was also opened. At the end, large impacted stone was also extracted (Figure 2). Stent was removed. Irrigation of common bile duct with saline was performed via this opening. There were no other stones. It was seen that there was one large opening between infundibulum, cystic duct, and CBD. A subtotal cholecystectomy was done. T-tube drainage was placed into the choledochus through this opening (Figure 3). By using the remaining part of the gallbladder, this opening was closed one by one with interrupted Ethicon 3-0 vicryl suture over this T-tube (Figure 4). Suture line was checked with injecting oliclinomel N4 Baxter (1000 mL) triple chambered parenteral nutrition solution through T-tube into the choledochus. Weak point was visualized by seeing white oliclinomel N4 coming out and reinforced by suturing this point again. After irrigation, one drainage tube was placed near the repaired part of choledochus. All the operation steps were summarized in illustration (Figure 5). Postoperative course was uneventful, total bilirubin level decreased from 4.8 to 1.0 mg/dL, and patient was discharged 7 days after the operation with T-tube. Final pathology (hematoxylin and eosin staining) was consistent with chronic cholecystitis. The T-tube was removed at the 21st day. At the 18-month follow-up, there was not any problem. He was symptom-free with normal liver function tests.

## 4. Discussion

Although many different treatment modalities are present, there is no consensus on the management of MS. Treatment of MS can be changed according to patient, type of MS, and experience of surgeon. If the patient has high risk for surgical intervention, endoscopic treatment can also be tried [10]. SC across Hartmann's pouch, infundibulum, or dilated cystic duct is considered to be a safe option in MS with aberrant and frozen anatomy within Calot's triangle where there is a potential risk of causing injury to the common bile duct (CBD) [11]. After SC, 3 main different surgical approaches are available for treatment of MS. The first option is closing the stump of gallbladder with suture or staple, the second option is the drainage of the remnant pouch by a drain or T-tube as in our case, and the third one is biliary drainage procedure with Roux-en-Y anastomosis to jejunum. The third option could be performed in 2 fashions as fistula-jejunostomy and hepaticojejunostomy and is suitable for MS types III and IV [6, 12, 13].

During MS treatment, open surgery is usually preferred. Laparoscopic surgery for MS remains controversial with most authors reporting high conversion rates with a range of 37–78% [14]. Many surgeons do not view conversion as detrimental and therefore do not persist on laparoscopy when cholecystectomy is difficult. As the level of MS increases (type I to type V), conversion rate increases concordantly. Piccinni et al. made an algorithm for management of MS and according to this algorithm MS > type II should be treated directly with laparotomy [6].

Antoniou et al. reported that laparoscopic surgery in MS is considered controversial and technically challenging, placing the patient at a probably unnecessary increased risk of bile duct injuries; as a consequence, laparoscopic cholecystectomy for MS cannot currently be recommended as a standard procedure [15]. Some authors also thought that it can be extremely challenging and time-consuming and may be associated with increased intraoperative and postoperative complications among these cases [16]. There is very rare reported data in the literature about laparoscopic treatment of a case with type III MS.

The most important thing during operation on a patient with MS is surgical decision which has to be changed according to the presence of aberrant and frozen anatomical structure. There were 3 important surgical steps, during laparoscopic operation on this patient with MS; the first one of them is SC, and the second one is extraction of impacted stone from cholecystocholedochal fistula via opening anterior surface of cystic duct. Irrigation of the choledochus with saline and exploring for other remaining calculi are also important through this opening. The third one is closing the opening on the anterior wall of choledochus with suture or staple. If no cholecystocholedochal fistula is identified, SC can be performed for type I MS. Surgical options in the presence of a cholecystocholedochal fistula include suture repair of the cholecystocholedochal fistula; choledochoplasty with the gallbladder remnant; endoscopic biliary stent placement; end-to-end anastomosis over a T-tube; and biliary enteric anastomosis [1].

After laparoscopic cholecystectomy in MS, complication rate of 0% to 60%, bile duct injury of 0% to 22%, and mortality ranging from 0% to 25% were reported in the literature [4]. These complications are classified as early and late. Main early complications are bile leakage, surgical infection, and bleeding. The late complications after surgical treatment of MS are stricture and remnant or recurrent stone formation in the remaining stump. The complication rate depends on the patient, type of MS, and type of applied surgery. After treatment of a patient with MS by SC and repair of choledochus with suturing over T-tube, bile leakage may be the most important early complication but it can resolve spontaneously with medical supportive treatment. In our case, bile leakage did not develop; it may be due to application of reinforcement suture in light of the checking with oliclinomel N4 injection at the end of the repair of stump. By this application, weak points of the repair were seen and reinforcement suture was applied over them. We thought that checking with oliclinomel N4 is safe and may decrease the bile leakage rate. After biliary tract repair or anastomosis operation, the most important late complication may be stricture formation at repaired part or Oddi sphincter. If it occurred, endoscopic and surgical management is not so easy to perform. Another late complication is the formation of residual gallstones in the remnant gallbladder. Symptomatic gallstone disease recurrence was reported to be 2.2%–5%. These complications can be treated successfully by endoscopic papillotomy or completion with cholecystectomy. Another one is gallbladder cancer which is found in 0.2–0.8% of patients undergoing laparoscopic cholecystectomy [12, 17]. In our case, either early or late complication developed during 18-month follow-up period.

## 5. Conclusion

Checking with oliclinomel N4 injection after biliary repair is safe and may decrease postoperative bile leakage rate. Laparoscopic SC, extraction of impacted stone, and repair of choledochus by suturing the remaining part of the gallbladder over the T-tube drainage can be applied safely with an experienced laparoscopic surgeon for treatment of type III MS.
